# *Plasmodium* gametocyte carriage in humans and sporozoite rate in anopheline mosquitoes in Gondar zuria district, Northwest Ethiopia

**DOI:** 10.1371/journal.pone.0306289

**Published:** 2024-07-01

**Authors:** Awoke Minwuyelet, Melkam Abiye, Ayalew Jejaw Zeleke, Sisay Getie

**Affiliations:** Department of Medical Parasitology, School of Biomedical and Laboratory Sciences, College of Medicine and Health Science, University of Gondar, Gondar, Ethiopia; Federal University of Agriculture, Abeokuta, NIGERIA

## Abstract

Although the overall burden of malaria is decreasing in Ethiopia, a recent report of an unpredictable increased incidence may be related to the presence of community-wide gametocyte-carrier individuals and a high proportion of infected vectors. This study aimed to reveal the current prevalence of gametocyte-carriage and the sporozoite infectivity rate of *Anopheles* vectors for *Plasmodium* parasites. A community-based cross-sectional study was conducted from May 01 to June 30/2019. A total of 53 households were selected using systematic random sampling and a 242 study participants were recruited. Additionally,515 adult female *Anopheles* mosquitoes were collected using Center for Diseases Control and Prevention (CDC) light traps and mouth aspirators. Parasite gametocytemia was determined using giemsa stain microscopy, while sporozoite infection was determined by giemsa staining microscopy and enzyme linked immunosorbent assay (ELISA). Among the total 242 study participants, 5.4% (95%, CI = 2.9–8.3) of them were positive for any of the *Plasmodium* species gametocyte. Furthermore, being female [AOR = 15.5(95%, CI = 1.71–140.39)], age group between 15–29 years old [AOR = 16.914 (95%, CI = 1.781–160.63)], no ITNs utilization [AOR = 16.7(95%, CI = 1.902 -146.727)], and high asexual parasite density [(95%, CI = 0.057–0.176, P = 0.001, F = 18.402)] were identified as statistically significant factors for gametocyte carriage. Whereas sporozoite infection rate was 11.6% (95%, CI = 8.2–15.5) and 12.7% (95%, CI = 9.6–16.3) by microscopy and ELISA, respectively. Overall, this study indicated that malaria remains to be an important public health problem in Gondar Zuria district where high gametocyte carriage rate and sporozoite infection rate could sustain its transmission and burden. Therefore, in Ethiopia, where malaria elimination program is underway, frequent, and active community-based surveillance of gametocytemia and sporozoite infection rate is important.

## Introduction

Although the largest reduction (by 40%) of mortality due to malaria was reported in Africa in 2017 compared to 2010, the overall malaria mortality reduction rate has slowed since 2015. The African Region remains home to the highest number of malaria cases and deaths. In 2022, an estimated 233 million malaria cases and 580, 000 deaths occurred in the African Region which accounted for about 94% of the cases and 95.4% of the deaths globally. *Plasmodium falciparum* (*P*. *falciparum*) is the most prevalent malaria parasite which accounts for 91.8% of estimated malaria cases [1].

Similarly, despite a remarkable reduction of malaria was shown in the last two decades, malaria remains a serious public health and socioeconomic burden in Ethiopia. For example, a statistic in 2019 revealed that malaria cases had decreased from 5.5 million to below 1 million. Likewise, the number of deaths has fallen from 3,000 in 2010 to 212 in 2021. However, according to the District Health Information Surveillance 2 (DHIS2) reported in 2021, malaria cases in Ethiopia increased by 55% compared to the 2019 report [2]. Likewise, in the first quarter of 2022 alone, there were 253,056 malaria cases that was higher than the number of cases reported in 2021 in the same period. This has been linked mostly to the interruption of malaria intervention programs in Afar, Amhara, and Tigray regions of the country due to extensive local unrest, the Covid-19 pandemic, and the introduction of a new malaria vector called *Anopheles stephensi (An*. *stephensi)* [3].

Malaria is transmitted by the bite of *Plasmodium*-infected female *Anopheles* mosquitoes. When these mosquitoes take a blood meal from a human, they inject sporozoites along with their saliva. Conversely, malaria is transmitted from humans to mosquitoes through the uptake of blood containing sexual stages of the parasite known as gametocytes, which develop from asexual stages within the infected human host. Several host factors have been linked to gametocyte development and gametocytemia, such as age, hematocrit/hemoglobin level, genotype, fever, and duration of symptoms [4, 5].

Although gametocytes of different *Plasmodium* species have varied developmental periods, only mature gametocytes are found in the peripheral blood circulation and are taken up by mosquitoes during blood feeding. The circulation time of gametocytes in infected individuals is influenced by the natural decay of gametocytes, anti-gametocyte immunity, and gametocytocidal drugs. Prolonged and persistent asymptomatic malaria infections, especially in endemic areas, are thought to promote gametocytogenesis and become a significant source of malaria transmission [5–7]. Therefore, gametocyte carriage rate can be used as an estimate of the transmission potential of infected individuals in areas where malaria is a public health problem.

Among the several female Anopheles mosquito species, 70 are known to transmit malaria parasites to humans. Of these, *Anopheles gambiae senso lato* and *An*. *funestus* are the predominant vectors globally [8–10]. In Africa, *An*. *gambiae senso stricto*, *An*. *arabiensis*, and *An*. *funestus* are the principal malaria vectors, with others like *An*. *pharoensis* and *An*. *coustani* also transmitting malaria in a few countries [9, 11–14].

Whereas *An*. *arabiensis*, a member of the *An*. *gambiae s*. *l* is the primary malaria vector in Ethiopia. In addition, *An*. *pharoensis*, *An*. *funestus*, and *An*. *nili* are also important malaria vectors in a few areas of the country [15–17]. In Ethiopia, transmission of malaria is highly seasonal and varies from place to place and year to year. Peak malaria transmission occurs between September and December in most parts of the country, which coincides with the planting and harvesting season. Whereas some areas of the country may experience a second minor malaria transmission period from April to June [15, 17]. Of interest, domestic travel was reported to be accountable for about 20% of the total cases of malaria in certain areas of the country [18] culminating that in synchrony with locally existing anopheles vectors, domestic imported malaria unless early management of cases takes place can contribute to about 20% of the local malaria transmission.

The current Ethiopian malaria control and elimination strategy includes using artemisinin-based combination therapy (ACT) as the first-line treatment for uncomplicated *P*. *falciparum* and chloroquine (CQ) for *P*. *vivax*, along with indoor residual spraying (IRS) and long-lasting insecticide-treated nets (LLITNs) [19, 20]. However, recent reports indicate an increasing incidence of malaria, suggesting the presence of factors undermining the national elimination strategy [15].

Given that Ethiopia is one of the tropical countries where the impact of global warming and therefore frequent change of climatic conditions is a significant problem, alongside propagation of climate adaptive malaria vectors, and it is suggested to be a challenge and become a threat to malaria control and elimination strategies [21]. Moreover, in low malaria-endemic areas especially during seasons where its transmission is very low, submicroscopic infection is common and contributes to 20–50% of the total malaria transmission [22]. In general, the prevalence of malaria in Ethiopia is estimated to be low or reduced which can be as high as 20% [23, 24]. Conversly, delayed treatment seeking is common and contributes to chronic asymptomatic infections that serve as significant sources of vector infection [25]. However, this contributes to the progression of chronic asymptomatic malaria infections that serves as a significant sources of malaria vector infection [26]. In sum, the presence of both human reservoir of the infection and conducive malaria transmitting anopheles vectors plays a synergetic role for sustained transmission of malaria across the country. In line, it had been reported that in malaria endemic areas status of human malaria reservoir may appear to be similar regardless of altitudes but the existence of suitable malaria transmitting vector is indispensable [27]. Therefore, continuous assessment of both human malaria reservoir and composition of sporozoite infected anopheles vector may play a significant role in forecasting future malaria incidences and to plan necessary intervention measures in support of Ethiopian malaria elimination program. Accordingly, we hypothesized that the prevalence of gametocyte carriers in communities and sporozoite carrier anopheles vectors attribute to sustain burden of malaria in Ethiopia. Therefore, this study was conducted to provide a current data about the gametocyte carriage and sporozoite detection rate in Gondar zuria district, Amhara, Ethiopia.

## Materials and methods

### Study setting and population

The research was carried out in Gondar Zuria district Northwest Ethiopia from May to June 2019. This district is located about 45 km from Gondar town and 686 km from Addis Ababa. Gondar Zuria District covers an area of 1,108.53 km^2^ and has a population density of 188.4 persons per km^2^ [28]. The district’s altitude ranges between 1,750 and 2,600 meters above sea level, and it is situated just north of Lake Tana. In 2018/2019, the total rainfall in the study area was 1,047.6 mm, with a mean maximum temperature of 27.4°C, a mean minimum temperature of 14.7°C, and a relative humidity of 45% [29].

A community-based cross-sectional study was conducted to assess the gametocyte carriage rate among community inhabitants, while a cross-sectional entomological survey was carried out to determine the sporozoite infection rate among the common Anopheles species known to transmit malaria in the country. Participants older than six months who had been living in the village for at least six months were included in the study, whereas individuals who were taking antimalarial drugs a month prior to and during data collection were excluded.

### Sample size determination and sampling technique

The minimum number of study participants was estimated by using a 7.73% prevalence (p) of gametocytes elsewhere in Ethiopia [30]. N = (Z_α/2_)^2^p (1-p) x DEF/d^2^: where n = the sample size, Z_α/2_ = 1.96 at 95% confidence level (Cl), DEF = design effect, d = margin of error at 5% (standard value of 0.05); and to account for dropout (missing) from village during actual data collection, 10% of the calculated sample size were added. Accordingly, the total sample size for gametocyte carriage detection was calculated to be 242, provided that the total number of individual study subjects was 242. To recruit study subjects two villages, among the 44 villages in the Gondar Zuria district, were selected randomly by a lottery method. The estimated numbers of households recruited for the study were proportionally allocated to the selected villages based on the existing household number using recent registration lists of households found at Health Post. Then, households were picked systematically considering the sample size required for the study, total number of households in the villages and the approximate family size per household. Accordingly, every individual in the selected households were enrolled as the study subject.

### Data collection and laboratory methods

A semi-structured questionnaire, which was prepared in English and translated to the local vernacular language (Amharic), was used to collect the socio-demographic factors like sex, age in years, marital status, educational status of participant father/mother, and household size of study participants. In addition, study relevant information on the *Anopheles* vectors like mosquito breeding habitat around the household and mosquito prevention methods such as bed net ownership of the household, ITNs utilization, history of IRS, previous history of malaria, treatment of previous history of malaria, and relapse history of the study participants were collected by trained local health extension workers along with the presence of the first author.

#### Laboratory sample collection and procedure

A capillary blood sample was taken aseptically, and a blood film was made and dried. Then, the blood films were transported to the Department of Medical Parasitology Laboratory, University of Gondar and stained with 10% fresh giemsa stain solution for 15 minutes, on the same day of sample collection. After the stained slides were air-dried, both thick and thin blood films were examined by the first author at 100X objective lens for the detection and identification of *Plasmodium* parasite (both asexual and gametocyte stages). A slide was considered negative if no *Plasmodium* parasite was seen after examination of 200 fields using the 100X objective lens. Gametocyte and asexual parasite densities were determined against 500 and 200 leukocytes, respectively, assuming a standard mean white blood cell count of 8,000 leukocytes per μl of blood [31]. A couple of slides were randomly selected and reexamined by the other co-authors to confirm consistency of detection of the *Plasmodium* parasite is maintained throughout the data collection. In addition, to assure the accuracy of malaria parasite detection and species identification and quantification, randomly selected slides were read by a qualified malaria microscopist who was certified by external agency that works on malaria research program. Blood film readings found to be discordant were reread by the first author and a value close to the qualified malaria microscopist was considered for the analysis.

#### Hemoglobin measurement

Finger-pick blood sample was used to measure hemoglobin concentration using a portable spectrophotometer (Haemocue 301). Anemia was classified as anemic and nonanemic based on the concentration of hemoglobin (Hgb). Accordingly, Hgb less than 11g/dl for pregnant women and children under 5 years old; Hgb less than 11.5g/dl for children with 5–14 years old; Hgb less than 12g/dl for non-pregnant women with age greater than 15 years old; and Hgb less than 13 g/ dl for men were considered as anemic [32].

#### Body temperature measurement

Axillary body temperature of the study participants was collected using thermometer reading. Study subjects were classified as febrile for axillary temperature reading greater than 37.5°C and non-febrile for axillary temperature reading less than 37.5°C.

#### Mosquito collection and identification

The CDC light traps and mouth aspirators were used to collect anopheles mosquitoes. For indoor mosquitos’ collection, CDC light trap was used in each randomly selected 10 houses in each village. The trap was installed on a wall at a height of 1.5 meter from the ground in the evening at 6:00 PM. At the same time, for the outdoor mosquito collection, another CDC light trap was suspended at the same height overnight [33, 34]. The CDC light traps were removed early in the morning and mosquitoes were collected. These procedures were repeated for six days (meaning six nights) in each of the selected houses during the two months of data collection period. Likewise, a mouth aspirator was used in a randomly selected houses in each village and mosquito collection was done in the early morning before the house opened. Then, the mosquitoes were placed into a labeled paper cup. All collected mosquitoes were transported to the Department of Medical Parasitology Laboratory, University of Gondar for storage and further analysis. Mosquitoes were killed by using chloroform, and the species of female *Anopheles* mosquitoes were identified according to the morphological characteristics [35].

*Anopheles* mosquitoes’ abdominal stage (unfed, freshly fed, half-gravid, and fully gravid) and parity were identified. The method used to determine parity in mosquitoes was the examination of the ovaries using microscopy. This is typically done depending on the stage of blood digestion and egg development (i.e., the gonotrophic stage) and through dissection of the mosquito under a microscope. The ovaries of mosquitoes allow for visually distinguishing between parous and nulliparous females [33, 34].

#### Dissection of mosquito’s salivary glands

Only parous *An*. *gambiae s*. *l* was dissected to examine sporozoite infection using giemsa stain microscopy. The remnant of dissected and all other undissected anopheles mosquitoes were kept in eppendorf tube at -20°C for further sporozoite detection using sandwich ELISA. Parous *An*. *gambiae* complex mosquitos were placed on a clean microscope slide and gentle pressure was applied to the thorax to squeeze the salivary glands into the neck. While gently pressing the thorax, a dissecting needle was used to pull the head of the mosquito in such a way that the salivary glands could be pulled out of the thorax. Then, salivary glands were detached from the head and a drop of physiological saline was placed on the salivary glands to keep the salivary glands from drying out as well as to maintain the tissues in a normal state for observation under a microscope. The fresh unstained salivary glands were examined for the presence of moving sporozoites under a 40x objective lens. Later, air-dried salivary glands were then fixed with 90% methanol and stained with 5% giemsa for 40 minutes. Finally, the percentage of mosquitoes infected with the sporozoites were calculated to determine the sporozoite infection rate [4, 36, 37]. Then, the sporozoite infection rates were scored according to the number of sporozoites observed: 1+ (1–10 sporozoites), 2+ (11–100 sporozoites), 3+ (101–1000), and 4+ (>1000 sporozoites) [38].


$$Infectivity\;Rate=\frac{Number\;of\;mosquitoes\;carrying\;sporozoites\;X100}{Number\;of\;mosquitoes\;dissected}$$

#### Mosquitoes processing for Circum Sporozoite Protein (CSP) detection

Individual *anopheles* mosquitoes were examined for CSP by using sandwich ELISA [39]. The thorax and head parts of female *Anopheles* mosquitoes were grounded in eppendorf tube by using a glass pestle and homogenized in 250ul of grinding buffer (that contains 0.5% IGEPAL CA-630 and 0.5% casein in phosphate buffer saline at pH 7.4). Sample homogenates along with positive and negative controls were titrated into a 96-well microtiter plate, coated with anti-*P*. *falciparum* and ant*-P*. *vivax* monoclonal antibodies (“Pv-210 and Pv-247”), and incubated for 30 minutes at room temperature [40]. Captured CSP antigen was revealed by a monoclonal antibody conjugated with horseradish peroxidase. The color intensity was measured, immediately after 30 minutes of incubation, using a spectrophotometer at 405nm. The sample absorbance higher than the cutoff value determined by using negative controls were considered as positive.

### Data quality control

To maintain the quality of data, data collection tools were pre-tested in one of the villages of the study area which later did not participate in the actual data collection. Test procedures were performed following manufacturer standards. Each questionnaire was coded, and any errors that appeared in the electronic database were cross-checked with the original questionnaire and data collection form.

### Data management and analysis

The data was appropriately coded and entered to an epi-data version 3.1 and transferred to the statistical package for social sciences (SPSS) version 23 software and checked for completeness and cleanness before analysis. Standard methods for the analysis of epidemiological data were used. Bivariable and multivariable analysis of factors associated with gametocyte carriage was conducted using logistic regression. Associated factors for gametocyte carriage that showed a P-value ≤ 0.2 in the bivariable analysis were selected and entered for multivariable logistic regression. Linear regression was used to evaluate the relationship between gametocyte and parasite density. A *P-value* < 0.05 was considered statistically significant.

### Ethical consideration

Ethical clearance: Ref. No. SBLS/2118/11 was obtained from the Ethical Review Committee of the School of Biomedical and Laboratory Sciences, College of Medicine and Health Sciences, University of Gondar. Permission from the Gondar zuria district Health Office and Village administration was also obtained. Before the study was conducted, Verbal informed consent was obtained from study participants above the age of 18 years old while permission and assent from parents/legal guardians were also obtained for study subjects below the age of 18 years of old. Study participants who tested positive for malaria were linked to nearby health institutions to get treated according to the National Malaria Diagnosis and Treatment Guidelines.

## Results

### Socio-demographic and clinical characteristics of the study participants

A total of 242 study participants, 131 from Tachtseda village and 111 from Hamsafeg village were included in this study. The majority of the study participants were females, 53.7% (130/242). The age of the study participants ranged from 9 months to 82 years with a mean of 24.67 (±21SD) years, and 60.4% (32/53) households’ heads were unable to read and write. Among the total of 53 households, 90.6% (48/53) owned bed net. However, out of the 242 study participants, only 66.9% (162/242) had daily sleeping habit under ITNs. In this study, 62% (150/242) of the study subjects had a previous history of malaria infection and nearly all had ever taken antimalarial drugs for their last malaria cases, of which 59.3% (89/150) of them had a history of relapse. Indoor residual spraying was sprayed five years ago in Tachtseda village whereas it was sprayed within the last 12 months in Hamsafeg village.

Whereas the mean hemoglobin concentration of the study participants was 13.14 (±1.43SD) g/dl which ranged from 8.30–17.90 g/dl. Similarly, the mean temperature of study participants was 35.7(± 0.94)°C which ranged from 32.10–38.70°C. Of the total study participants involved, 16.1% (39/242) of them were anemic and 2.5% (6/242) of them were febrile (**Table 1**).

**Table 1:** Association of gametocyte carriage with sex, age group, asexual parasitemia density, hemoglobin concentration, and axilliary body temperature among study participants in Gondar zuria district, Northwest Ethiopia, 2019.

Variables	Gametocyte carriage		χ2	P-value
	Yes [n (%)]	No [n (%)]		
**Sex**				
Male	3 (2.7)	109 (97.3)	2.071	0.15
Female	10 (7.7)	120 (92.3)		
**Age in years**				
<5	2 (6.5)	29 (93.5)	8.277	0.041
5–14	2 (2.5)	77 (97.5)		
15–29	7 (13.7)	44 (86.3)		
≥30	2 (2.5)	79 (97.5)		
**Asexual parasite density**				
< 500 parasite /ul	6 (26.1)	17 (73.9)		0.001
≥ 500 parasite /ul	7 (100)	0		
**Hemoglobin level**				
Anemic	6 (15.4)	33 (84.6)		0.009
Non-anemic	7 (3.4)	196 (96.6)		
**Body temperature**				
<37.5°C	12 (5.1)	224 (94.9)		0.285
≥37.5°C	1 (16.7)	5 (83.3)		
**Villages**				
Tachetseda	8 (6.1)	123 (93.9)	0.07	0.791
Hamsafeg	5 (4.5)	106 (95.5)		

### Prevalence of gametocyte carriage

Among the total 242 study participants, the overall prevalence of malaria was 12.4%, (30/242, 95%, CI = 8.7–16.5). Of those found to be infected, *P*. *falciparum* and *P*. *vivax* infections accounted for 16.7% (5/30) and 83.3% (25/30), respectively. The majority [86.7%, (26/30)] of infected subjects were asymptomatic except 16% (4/25) of *P*. *vivax* infections which were febrile.

Whereas the overall gametocyte carriage prevalence was 5.4% (13/242, 95% CI = 2.9–8.3). Infection due to *P*. *vivax* contributed to 3.7% (9/242) of the overall gametocyte prevalence while *P*. *falciparum* contributed 1.7% (4/242). Of note, among the total infected study participants, gametocyte infection was detected in 43.3% (13/30) of the cases. Furthermore, *P*. *vivax* gametocyte infection contributed to 30% (9/30) of the total cases of malaria while *P*. *falciparum* accounted for 13.3% (4/30) of the total cases.

In this study, a higher gametocyte carriage was found among female study participants (4.3%, 10/242). Likewise, the age group 15–29 years old contributed to the higher proportion of gametocytes carriage rate 2.9% (7/242). Conversely, 15–14 years old and over 30 years-old participants had the lowest gametocyte infection rate. Of the two participating villages, the majority (3.3%, 8/242) of gametocyte carriage rate was found in Tachtseda village. Overall, this study revealed that there was a statistically significant association between age group and gametocyte carriage rate (Chi-square, likelihood ratio, χ2 = 8.277, P = 0.041) (**Table 1**).

Moreover, in this study among the total study participants, 2.5% (6/242) of them who were found to be *Plasmodium* gametocyte-infected were anemic. Likewise, of the total anemic study participants, 15.4% (6/39) of them had gametocytemia. Moreover, among the total study participants, 2.5% (6/242) of them had asexual parasite density below 500parasites/ul and gametocytemia. Alternatively, of the total study participants who had a parasite density below 500parsites/ul, 26.1% (6/23) of them had gametocytemia. Unfortunately, all study participants who had asexual parasite density greater than 500 parasites/ul had gametocytemia. Asexual parasite density had a significant association with gametocyte carriage rate (Chi-squar test, fisher’s exact test, P = 0.001) (**Table 1**). However, due to the co-existence of asexual parasitemia and gametocytemia, perhaps the latter depends on the former, the association between anemia and gametocytemia may require careful interpretation.

### Bivariable and multivariable analysis of associated factors with gametocyte carriage

Factors including sex, age, bed net usage, hemoglobin level, and asexual parasite density were significantly associated with gametocyte carriage rate (*P<0*.*05*). For example, being females were 15.5 times more likely to carry gametocytes than those males [AOR = 15.5 (95%, CI = 1.71–140.39)]. Similarly, the odds for 15–29 years old study participants to develop gametocyte carriage rate were about 17 times more likely than those of ≥30 years old [AOR = 16.914 (95%,CI = 1.781–160.63)]. Additionally, individuals who did not sleep under ITNs had 16.7 times more likely to carry gametocyte than those who slept under ITNs daily [AOR = 16.7(95%, CI = 1.902–146.727)]. This study has also illustrated that individuals who were anemic were 8.758 times more likely to harbor gametocyte infection than those who were non-anemic participants [AOR = 8.758 (95%, CI = 1.37–56.007)] (**Table 2**). Moreover, this study revealed that asexual parasite density and gametocytemia had a strong positive relationship (r^2^ = 0.791, P = 0.001). Accordingly, a one-unit increase in asexual parasitemia tends to increase gametocytemia by 0.057 times [95% CI = 0.057–0.176, P = 0.001, F = 18.402)].

**Table 2:** Bi-variable and multi-variable analysis of associated factors for gametocyte carriage rate, Gondar zuria district, Northwest Ethiopia, 2019.

Variables	Gametocyte carriage			OR (95%,CI)		
	Pos (%)	Neg (%)	Total (%)	COR	AOR	*P value*
**Sex** Male	3 (2.7%)	109(97.3)	112(46.3)	1	1	
Female	10(7.7)	120(92.3)	130(53.7)	3.028(0.818–11.289)	15.5(1.71–140.39)	0.015
**Age in years**						
<5	2 (6.5)	29(93.5)	31(12.8)	2.724(0.367–20.242)	0.34(0.2–7.13)	0.519
5–14	2(2.5)	77(97.5)	79(32.6)	1.026(0.141–7.468)	3.35(0.35–31.965)	0.293
15–29	7(13.7)	44(86.3)	51(21.2)	6.284(1.251–31.568)	16.914(1.781–160.63)	0.014
≥30	2(2.5)	79(97.5)	81(33.5)	1	1	
**Family education**						
Illiterate	9(6.1)	139(93.9)	148(61.8)	0.906(0.107–7.688	------	0.928
Read and write	3(3.8)	76(96.2)	79(32.6)	0.553(0.054–5.702		0.628
≥ Primary school	1(6.7)	14(93.3)	15(6.2)	1		
**Availability of ITNs**						
Yes	9(4,1)	211(95.9)	220(90.9)	0.192(0.054–0.684	0.44(0.05–3.856)	0.459
No	4 (18.2)	18(81.8)	22(9.1)	1	1	
**Utilization of ITNs**						
Daily	2(1.2)	160(98.8)	162(66.9)	1	1	
Occasionally	2(6.5)	29(93.5)	31(12.8)	5.517(0.747–40.746)	7.43(0.574–96.205)	0.125
Not at all	9(18.4	40(81.6)	49(20.2)	18(3.742–86.594)	16.7(1.902–146.727)	0.011
IRS the last 12 months						
Yes	3(2.9)	102(97.1)	105(43.4)	0.374(0.1–1.394)	0.321(0.043–2.428)	0.271
No	10(7.3)	127(92.7)	137(56.6)	1	1	
mosquito breeding site from house						
≤1 km	8(5.4)	141(94.6)	149(61.6)	0.999(0.317–3.149)	-----	0.998
>1 km	5(5.4)	88(94.6)	93(38.4	1		
**Family ever sick by malaria before**						
Yes	10(6.7)	140(93.3)	150(62)	2.119 (0.568–7.911)	-----	0.264
No	3(3.3)	89(96.7)	92(38)	1		
**Treated previous malaria cases**						
Yes	10(6.9)	134(93.1)	144(59.5)	2.363(0.633–8.817)	----	0.2
No	3(3.1)	95(96.90	98 (40.5)	1		
**Relapse history**						
Yes	3(3.4) 7(11.5)	86(96.7)	89(59.3)	0.269(0.067–1.085)	0.283(0.05–1.599)	0.153
No		54(88.5)	61(40.7)	1	1	
**Hemoglobin level**						
Anemic	6(15.4)	33(84.6)	39(16.1)	5.091(1.61–16.096)	8.758(1.37–56.007)	**0.022**
Non-anemic	7(3.4)	196(96.6)	203(83.9)	1	1	
**Body temperature**						
<37.5	12(5.1)	224(94.9)	236(97.5)	0.268(0.29–2.477)	----	0.246
≥37.5	1(20)	5(8)	6(2.5)	1		

Pos:Postive, Neg: Negative

### Species composition and abundance of *Anopheles* mosquitoes

A total of 515 adult female *Anopheles* mosquitoes were collected from the two villages by using CDC light traps and mouth aspirators collection techniques. Six *Anopheles* species including *An*. *gambiae s*. *l*, *An*. *pretoriensis*, *An*. *cinereus*, *An*. *longipalpis*, *An*. *Azaniae*, and *An*. *nilli* were identified. From the total female anopheles mosquitoes collected, *An*. *gambiae* complex was the predominant species [97.3% (501/515)] followed by *An*. *pretoriensis* 1.5% (8/515) (**Table 3**).

**Table 3:** Species composition and number of *Anopheles* mosquitoes collected by two different methods in Gondar zuria district, Northwest Ethiopia, 2019.

Study sites		CDC light traps collections		Aspirators	Total (%)
	Species	Indoor (%)	Outdoor (%)	Mouth aspirator (%)	
**Tachtseda**	*An*.*gambiae s*.*l*	231(97.8)	74(94.8)	61(95.3)	366(96.8)
	*An*. pretoriensis*	3(1.3)	2(2.6)	1(1.6)	6(1.6)
	*An*.cinercus*	2(0.8)	0	0	2(0.5)
	*An*.azaniae*	0	2(2.6)	0	2(0.5)
	*An*. longipalpis*	0	0	1(1.6)	1(0.25)
	*An*.nilli*	0	0	1(1.6)	1(0.25)
	Total	236(100)	78 (100)	64(100)	378(100)
**Hamsafeg**	*An*.*gambiae s*.*l*	79 (97.5)	34(100)	22(100)	135(98.5)
	*An*.pretoriensis*	2(2.5)	0	0	2(1.5)
	Total	81(100)	34 (100)	22 (100)	137(100)

*Indicates the need for further investigation to confirm species identification

In this study, three hundred seventy-eight females anopheles mosquitoes (73.4%) were collected from the village where there was no IRS coverage in the last 5 years and practicing small irrigation (Tachtseda), while the rest 137 (26.6%) were collected and identified in the human settlement village near to the river (Hamsafeg). However, there was no statistically significant difference in the distribution of anopheles mosquitoes between two the villages (Chi-squar test, likely hood ratio, χ2 = 3.752, df = 5, P = 0.586).

### Sporozoite detection rate

Among the total 515 anopheles mosquitoes collected, we were able to analyze 386 of them for the sporozoite detection using giemsa stain microscopy or ELISA. The proportion of parity of overall *An*. *gambiae s*. *l* was 73%. For the giemsa stain microscopy detection, only parous *An*. *Gambiae s*. *l* were used. Accordingly, we analyzed 309 of the *An*. *gambiae* s. l for microscopy detection and the overall sporozoite infectivity rate was 11.6% (36/309) at (95%, CI = 8.4–15.2). Among the total infected anopheles mosquitoes examined with giemsa microscopy, 52.78% of them were plus two, 44.44% of them were plus one, and only 2.78% of them were plus three for sporozoite infection rate.

Whereas we analyzed 386 individual anopheles mosquitoes, including those examined microscopically, using ELISA for the detection of CSP and species identification. Accordingly, CSP was detected only in 12.7% (49/386) of the anopheles mosquitoes and all of them were the *An*. *gambiae s*. *l*. whereas of those CSP ELISA-positive mosquitoes samples, *P*. *falciparum* and

*P*. *vivax* sporozoites infections accounted for 8.1% (4/49) and 91.8% (45/49), respectively. Furthermore, among microscopic positive anopheles mosquitos’ samples, 22.2% (8/38) of them turned negative by ELISA. Conversely, among the total microscopic negative samples, 4.8 (13/273) of them appeared to be positive by ELISA (Table 4).

**Table 4:** Sporozoite detection rate by Giemsa microscopy and ELISA in Gondar zuia district, Northwest Ethiopia, 2019.

*Anopheles* species	Microscopic detection (N)	ELISA detection (N)
*An*. *gambiae s*. *l* (used for microscopic examination)	Positive (36)	Positive (28)
		Negative (8)
	Negative (273)	Positive (13)
		Negative (260)
Undissected *An*. *gambiae s*. *l*	Non applicable	Positive (8)
		Negative (56)
*An*. *Pretoriensis*	Non applicable	Positive (0)
		Negative (8)
*An*. *cinereus*, *An*. *longipalpis*, *An*. *Azaniae*, *and An*. *nilli*	Non applicable	Positive (0)
		Negative (5)

## Discussion

Malaria is endemic in Ethiopia and deteriorates the health and socioeconomic conditions of majority of its people. As a result, to harness the fight against malaria Ethiopia is currently adopting a strategy that emphasizes the need for an improved focus of malaria transmission reduction that lead to elimination, implying that active community wide detection of *Plasmodium* gametocyte carriage and anopheles vector sporozoite infectivity play indispensable rolegram. This is because, in areas where malaria is sustained at low levels or is highly seasonal, asymptomatic reservoirs of the infection are critical for maintaining its transmission [5].

In the current study, the overall prevalence of gametocyte carriage was 5.4% (95%, CI = 2.9–8.3). This finding was comparable with the studies conducted elsewhere in northwest Ethiopia [41], southwestern Ethiopia [30] and western Thailand [42] which revealed gametocyte carriage rate of 3.3%, 7.73% and 3.3%, respectively. However, it was higher than the studies conducted in south-central Oromia, Ethiopia 1.5% [43], Southern Zambia 2.3% [44]. This difference could be explained by variations in geographical locations (rainfall, temperature, and altitude), sample size, study period, the available malaria control programs in the study areas, and diagnosis tools used. Particularly, along with the inherent limitations of Giemsa microscopy in detecting *Plasmodium* parasites, competency of malaria microscopists to identify gametocytes stages would affect its overall prevalence. Likewise, a significant proportion of gametocyte carriage which ranges between 17–40% as identified by real-time quantitative PCR (RT-qPCR) were reported as negative by microscopy [45]. In this study, infection due to *P*. *vivax* contributed to 3.7% of the overall gametocyte carriage that was greater than gametocyte carriage (1.7%) due to *P*. *falciparum*. The low prevalence of *P*. *falciparum* gametocyte infection in the current study could be associated with its scarcity in highland areas of the country. In line, it had been suggested that the prevalence of *P falciparum* could be low or can have an inverse relationship with altitude [46]. Moreover, cytoadherence and parasite sequestration of the total *P*. *falciparum* may trigger development of gametocytes in visceral tissues and hindering its detection in peripheral circulation [5]. Conversely, a faster development of *P*. *vivax* gametocytes and blood distribution of all parasite stages of *P*. *vivax* may result a higher proportion of gametocyte carriage (51).

In addition, although small sample size was used, the current study revealed that the prevalence of gametocyte carriage among male subjects was lower (2.7%) than females (7.8%, P = 0.015). This finding was supported by a study conducted in Thailand [47]. However, it was found to be contrasting with other studies conducted in south-central Oromia, Ethiopia [43] and western Thailand [42]. The sexual distribution difference in gametocyte carriage between ours and previous studies could be associated with individuals’ differences with respect to their immunity, white blood cells count, and metabolism based on sex [48, 49]. For example, female patients were suggested to control malaria infection and can adapt especially the low parasitemia density than males [49], suggesting a high probability of gametocytemia among females. Whereas the prevalence of *Plasmodium* gametocytes carriage rate was significantly higher among study participants with the age group between 15 to 29 years old (13.7%) (P<0.05). Similar result was also reported in India [50], Tanzania [51], and Peru [52]. In contrast, others reported that a significantly higher prevalence of gametocytes was detected in younger children [53, 54]. However, existing finding implicated that adults with 15–29 years old could be a significant reservoir for malaria transmission [55]. Many factors might have accounted for the unique age patterns of parasitemia and gametocytemia observed in the present study. For example, the highest odds of parasitemia and gametocytemia in adults could be attributed from the low malaria control programs targeting adults than children and pregnant women and they were also having a poor practice of ITNs utilization. In support of this, elsewhere in Ethiopia, young adults in the age between 18 to 25 years were reported to have a poor treatment seeking behaviour [56]. Intriguingly, better clinical immunity among young adults, typical in malaria endemic areas, could also play several roles in maintaining low-grade asexual parasitemia and constant modulation of sexual development in young adults [26, 57].

Furthermore, in this study, hemoglobin concentration of infected individuals was identified as an important determinant for gametocyte carriage. Accordingly, anemic individuals were associated with higher odds of (8.758) gametocyte carriage than those non-anemic *Plasmodium* infected participants. This is supported by the study conducted in western Kenya [58], Southern Ghana [59], and Gambia [60]. It is still unclear to what extent mechanisms are involved in the relationship between anemia and gametocytogenesis. However, the possible reason might be that a longer duration of malarial infection may contribute to anemia. Nonetheless, due to anemia, the high production of reticulocytes could provide a favorable shelter and pathway of gametocytogenesis [61–64]. In addition, this study showed that the high density of parasitemia and gametocytemia had a strong relationship (r^2^ = 0.79). This finding is supported by studies conducted in western Kenya [65], Southern Ghana [59], and western Thailand [42], suggesting that higher parasite density might cause more frequent genetic recombination necessary for parasite replication [66].

Moreover, in the present study, gametocyte carriage and bed net utilization were highly associated. The odds of being infected and carrying gametocyte was higher in those individuals who were not using a bed net or only using it occasionally than those who used it daily (p<0.05). This finding is supported by studies conducted elsewhere in Northwest Ethiopia [67], in western Kenya [65] and in Southern Ghana [59]. ITNs protect against malaria thereby reducing transmission of malaria parasites [68]. In the current study, ownership of ITNs was 90.6%, though usage pattern differs among the study subjects. This difference could be explained by differences in the knowledge, attitude, and practice of individuals in different study areas towards frequent and appropriate usage of ITNs as well as the success of overall malaria control and prevention measures [56].Whereas this study revealed that greater number of *anopheles* mosquitoes were sampled from indoors, (61.7%) using CDC light traps and (16.7%) using mouth aspirators, than outdoors from the area where IRS was not performed in the last 12 months. This higher number of indoor collection agreed with a report from Lake Victoria, Kenya [69]. However, it appeared to be contradictory with reports in different parts of Ethiopia [70–72]. This variation could be accounted due to the difference in malaria control interventions and collection seasons. A study had shown that application of different intervention strategies makes different feeding and resting behaviour of malaria vectors [73]. Nonetheless, the absence of IRS in the last 12 months in the current study could supported more females anopheles mosquitoes to spent much time in the houses after feeding indoors [74].

In the present study, *Anopheles gambiae s*. *l* remains the dominant vector. Accordingly, the overall sporozoite infection rate in *An*. *gambaie s*. *l* was 11.6% (36/309) (95%, CI = 8.4–15.2) and 12.7% (49/386) at (95%, CI = 9.6–16.3) by microscopy and ELISA, respectively. This finding was comparable with the study conducted elsewhere in Ethiopia, 15.2% [72]. However, it was lower compared to others such as southwestern Ethiopia 17.1% [71] and central Nigeria 31.6% [37]. Conversely, the current result was higher compared to other findings from Sille, Ethiopia 2.6% [75] and southern Ethiopia 1.04% [76], suburbs of Jimma town, Ethiopia 1.8% [72], and central Ethiopia 1.18% [77], Kenya 1.8% [78], Yemen 0.9% [79] and Senegal 0.64% [80]. This variation could be attributed from anopheles vector ecological factors, data collection season, and sample collection techniques, use of ITNs, IRS coverage, and diagnostic technique used. Furthermore, although the sporozoite detection rate by microscopy (11.6%) was nearly similar with ELISA (12.7%) detection, there was discrepancy in sporozoite detection by diagnostic techniques (Table 4). Similar findings were observed in the Guinea [81] and western Kenya [8]. The discrepancy of detection of sporozoite between diagnostic tools might be associated with the limited sensitivity of microscopy and a reduced integrity of CSP due to the use of anopheles mosquitoes for microscopy, which underestimates performance of ELISA. Of interest, this study revealed that among the total microscopy negative results, 4.8% of them were positive by ELISA. Conversely, among the total microscopy positive results, 22.2% of them turned negative by ELISA. This can be associated with the concurrent inherent limitation of microscopy such as its low sensitivity as well as poor competency of malaria microscopist [45]. Similarly, a 78.8% higher sporozoite infectivity rate was reported in western Kenya by using ELISA than microscopy [9].

### Limitation of the study

The prevalence of sexual and asexual parasitic stage was determined solely by geimsa microscopic. Likewise, species identification of anopheles mosquitoes was determined with visual morphological examination of external physical features. In addition, the limited number of houses included the limited frequency of collection and limited methods of mosquito collection and short study period may underestimate the results of this study.

## Supporting information

S1 Data (XLS)

## Abbreviations

ACT: Artemisinin based combination therapy
CDC: Center for Diseases Control and Prevention
CSP: Circum sporozoite protein
ELISA: Enzyme-Linked Immunosorbent Assay
IRS: Indoor Residual Spraying
ITNs: Insecticide Treated Nets
